# Clarifying deprescribing

**DOI:** 10.1177/10398562241303398

**Published:** 2024-11-26

**Authors:** Niall McLaren

**Affiliations:** Brisbane, QLD

Dear Editor,

A recent 600-word letter^1^ on deprescribing briefly indicates that a large proportion of antidepressant prescriptions are inappropriate and suggests a range of public health measures as correctives (essentially, adhering to practice guidelines). Oddly enough, seven members of the editorial board of this journal objected to its conclusions and penned a 3300-word response.^2^ While clearly agreeing with the letter’s main points—that antidepressants are being prescribed at high and rising rates, often inappropriately, for too long and at unnecessary expense—they opposed standard measures to reduce prescription rates.

The editors claimed that antidepressant “… usage in health economics studies reveals patient preferences, which are for the prescription of antidepressants … Patient revealed preferences for antidepressant treatment inherent in the rising prescribing rate.” If, which I don’t concede, many patients ask for them, that reflects the pervasive effect of drug company marketing, not of their knowledge of pharmacology. The idea that patients should decide their own treatment is wholly unprofessional.

They said (p. 4) “‘health outcomes’ cannot be easily linked to interventions for mental illness…” Is this true? Surely the whole thrust of the case for drugs in mainstream psychiatry is that mental disorder is biological in nature and only drugs can and do produce adequate “health outcomes”? If there is no linkage, then why are there drug trials?

While agreeing that antidepressants have adverse consequences, including withdrawal effects,^3^ the editors tried to weaken the argument by saying this is also true of many forms of treatment. They further recycled this “objection” by saying other drugs are often prescribed inappropriately or for too long. That is not the issue: we are talking about antidepressants, not about other drugs. I cannot escape a speeding fine by pointing out that other drivers were speeding, too.

In concluding (p. 5), they said “…an a priori assumption that antidepressants are harmful, and that they need to be deprescribed (is) likely to do more harm than good.” This is a straw man. The letter did not state or imply that, but focuses instead on “… the human costs (and) substantial unnecessary economic costs…” of ill-advised prescribing (p. 1).

Finally, the editors state (p. 2): “We agree that not all antidepressant prescribing is warranted” but later concede: “… it remains unclear who these medications are being administered to and indeed why. Therefore, it is not possible to determine the appropriateness of the prescribing patterns” (p. 4). This admission destroys the editors’ case: 70 years after antidepressants were introduced, they still can’t say who gets them, why, or why they take them for so long, but they don’t want them reduced. At about $30 a script, 3.2 million Australian adults taking a year’s supply of the drugs costs over $1 billion. Surely psychiatrists owe it to the general public to know what they’re doing and why before taking such a strong position? Perhaps the editors could try proving that depression is a primary biological phenomenon demanding biological treatment, because nobody has ever done it.^4^
